# A new exceptionally well-preserved basal actinopterygian fish in the juvenile stage from the Upper Triassic Amisan Formation of South Korea

**DOI:** 10.1038/s41598-023-50803-z

**Published:** 2024-01-03

**Authors:** Su-Hwan Kim, Yuong-Nam Lee, Gi-Soo Nam, Jin-Young Park, Sungjin Lee, Minyoung Son

**Affiliations:** 1 School of Earth and Environmental Sciences, Seoul National University, Seoul, 08826 South Korea; 2 Gongju National University of Education, Gongju, South Chungcheong 32553 South Korea; 3 Gwacheon National Science Museum, Gwacheon-si, Gyeonggi-do 13817 South Korea; 4 Department of Earth and Environmental Sciences, University of Minnesota Twin Cities, Minneapolis, MN USA

**Keywords:** Evolution, Palaeontology

## Abstract

The study of the large paraphyletic group of extinct ‘palaeoniscoid’ fishes has shed light on the diversity and evolutionary history of basal actinopterygians. However, only a little ontogenetic information about ‘palaeoniscoids’ is known because their records in the early stages of development are scarce. Here, we report on a growth series of ‘palaeoniscoids’ in the juvenile stage from the Upper Triassic Amisan Formation of South Korea. Fourteen specimens, including five counterpart specimens, represent a new taxon, *Megalomatia minima* gen. et sp. nov., exhibiting ontogeny and exceptional preservation with the eyes possibly containing the crystalline lens, the otoliths, and the lateral line canals without covering scales. This discovery allows us to discuss the adaptations and evolution of basal actinopterygians in more detail than before. The otoliths in situ of *Megalomatia* support the previous interpretation that basal actinopterygians have a sagitta as the largest otolith. The trunk lateral line canal, which runs under the scales instead of passing through them, represents a plesiomorphic gnathostome trait. Notably, the large protruded eyes suggest that *Megalomatia* probably has binocular vision, which would have played a significant role in targeting and catching prey with the primitive jaw structure. In addition, the firstly formed skeletal elements such as the jaws, pectoral girdle, and opercular series, and the posteroanterior pattern of squamation development are likely linked to the adaptation of young individuals to increase their viability for feeding, respiration, and swimming.

## Introduction

The study of fish ontogenies provides comprehensive knowledge of their developmental patterns, functional development tendencies, and environmental preferences according to different developmental stages^1–3^. In addition, the characteristics observed in fish throughout their developmental stages reflect possible adaptations in response to functional demands, probably correlating with individual survival success. However, examining fossilized fish ontogenies is challenging because immature specimens are seldom mineralized^4^, and the period during which early developmental conditions are exhibited occupies a relatively small portion of the lifespan of an organism. Furthermore, to avoid misidentification of different ontogenetic stages of a species as different species, various criteria are required, such as small body size, degree of ossification and squamation, and body proportion^3,5^. For these reasons, the fossil record of fish ontogenies is scarce and mostly restricted to studying size changes and squamation development^4,6–8^.

**Figure 1:**
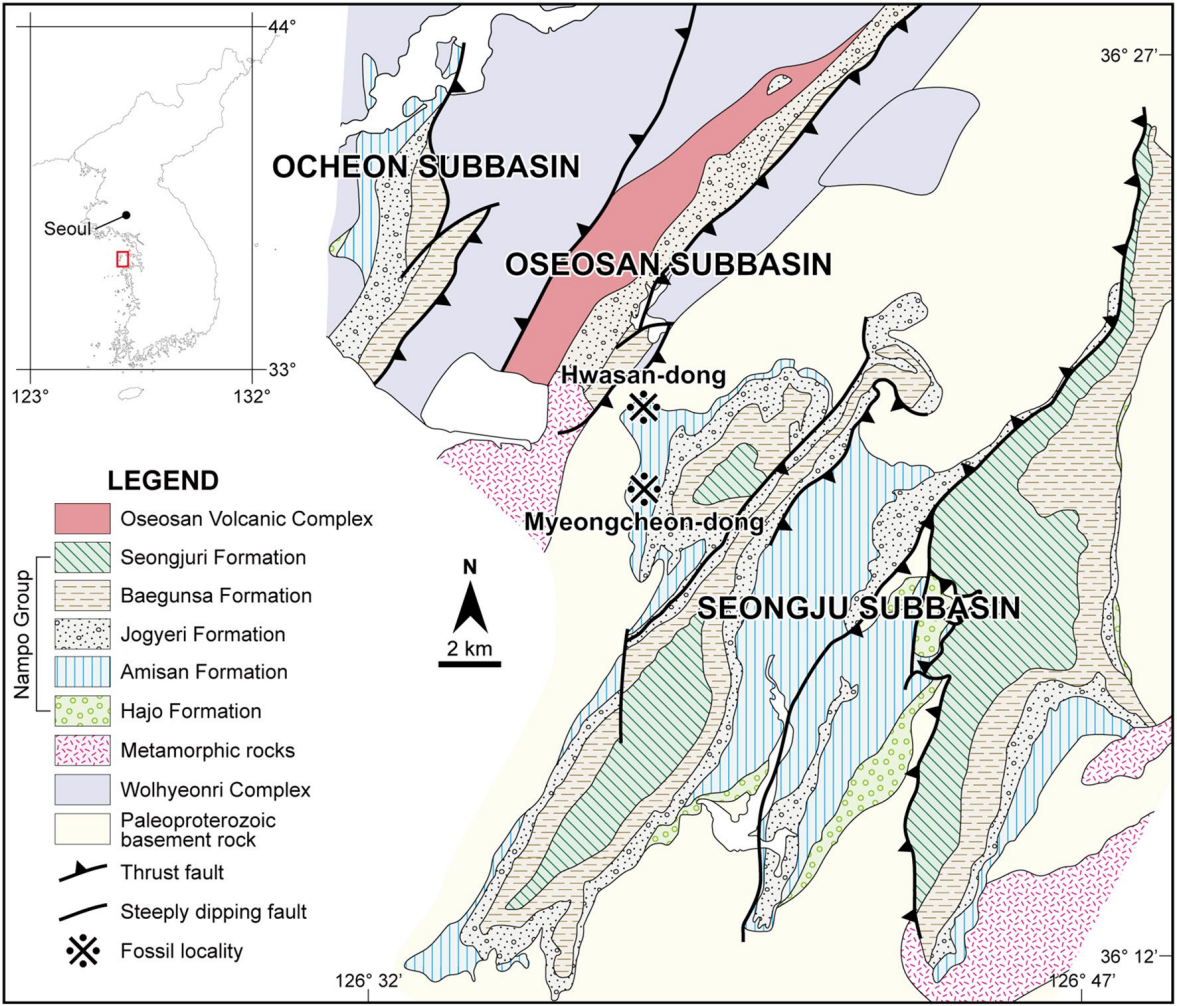
Geological map of the study area and locality where the specimens were discovered (modified from Egawa and Lee^9^; Park et al.^17^).

‘Palaeoniscoids’ are a paraphyletic assemblage of basal actinopterygians from the Late Silurian to the Cretaceous^9–11^. They are morphologically characterized by firmly united cheekbones, generally inclined hyomandibula, large and far forwardly positioned eyes, strongly heterocercal caudal fin, and rhombic scales with peg and socket articulations^9,12^. A new ‘palaeoniscoid’ fish, *Megalomatia minima* gen. et sp. nov., is described based on fourteen specimens, including five counterpart specimens from the Amisan Formation of South Korea (Fig. 1, blue stripes). The Amisan Formation is interpreted as alluvial-fan to lacustrine-delta environments^13^, and its depositional age is generally considered Late Triassic or Late Triassic-Early Jurassic based on fossil records^14,15^. A single specimen of Redfieldiiformes, *Hiascoactinus boryeongensis*, was reported from the Amisan Formation, and it is distinguished from *Megalomatia* mainly by a single plate-like branchiostegal ray and the caudally positioned dorsal and anal fins in near opposition to each other^16^. Accordingly, *Megalomatia* represents a new taxon, not different ontogenetic stages of *Hiascoactinus*. This study aims to report a new ‘palaeoniscoids’ with a description of its ontogeny and to examine the functional adaptations that enhance its viability.

## Materials and methods

Fourteen specimens, including five counterpart specimens, were collected from the Amisan Formation at Myeongcheon-dong and Hwasan-dong, Boryeong, South Chungcheong Province, South Korea (Fig. 1). They are preserved on the black shale as fossil slabs, showing lateral or ventral side. The holotype specimen (GNUE11001) was collected in 2008 from the Myeongcheon-dong section and is housed in the Gongju National University of Education (GNUE), Gongju, South Chungcheong Province, South Korea. The other specimens are collected from the Hwasan-dong section without collection dates and stored in the Boryeong Coal Museum (BCM), Boryeong, South Chungcheong Province, South Korea. Despite their relatively small size (less than 35 mm in standard length (SL), from the tip of the snout to the level of ventral origin of the caudal fin), the specimens show exceptional preservation of the whole body, including the eyes, possibly containing the crystalline lens, the otoliths, and the trunk lateral line canals without covering scales. The preserved organic residues are light brown in color. The specimens were examined using a stereo microscope (Leica M165C) and a digital camera (Sony A7R4A). The specimens were photographed with different lighting angles to highlight the relief of the surface, so the photographs of the specimens have slight differences in colors despite actually sharing the same color. The line drawing of the specimens was done using Adobe Illustrator. The meristic measurements of all specimens are recorded in Supplementary Table S1.

## Results

### Systematic paleontology

Class Osteichthyes Huxley^18^.

Subclass Actinopterygii Cope^19^.

Family *incertae sedis*

*Megalomatia minima* gen. et sp. nov.

#### Etymology

The generic name is derived from the Greek “megálo” (big) and the “mátia” (eyes), referring to the large eyes of the new specimen. The specific epithet “minima” (small in Greek) refers to the small body size of the new specimen.

#### Holotype

**Figure 2:**
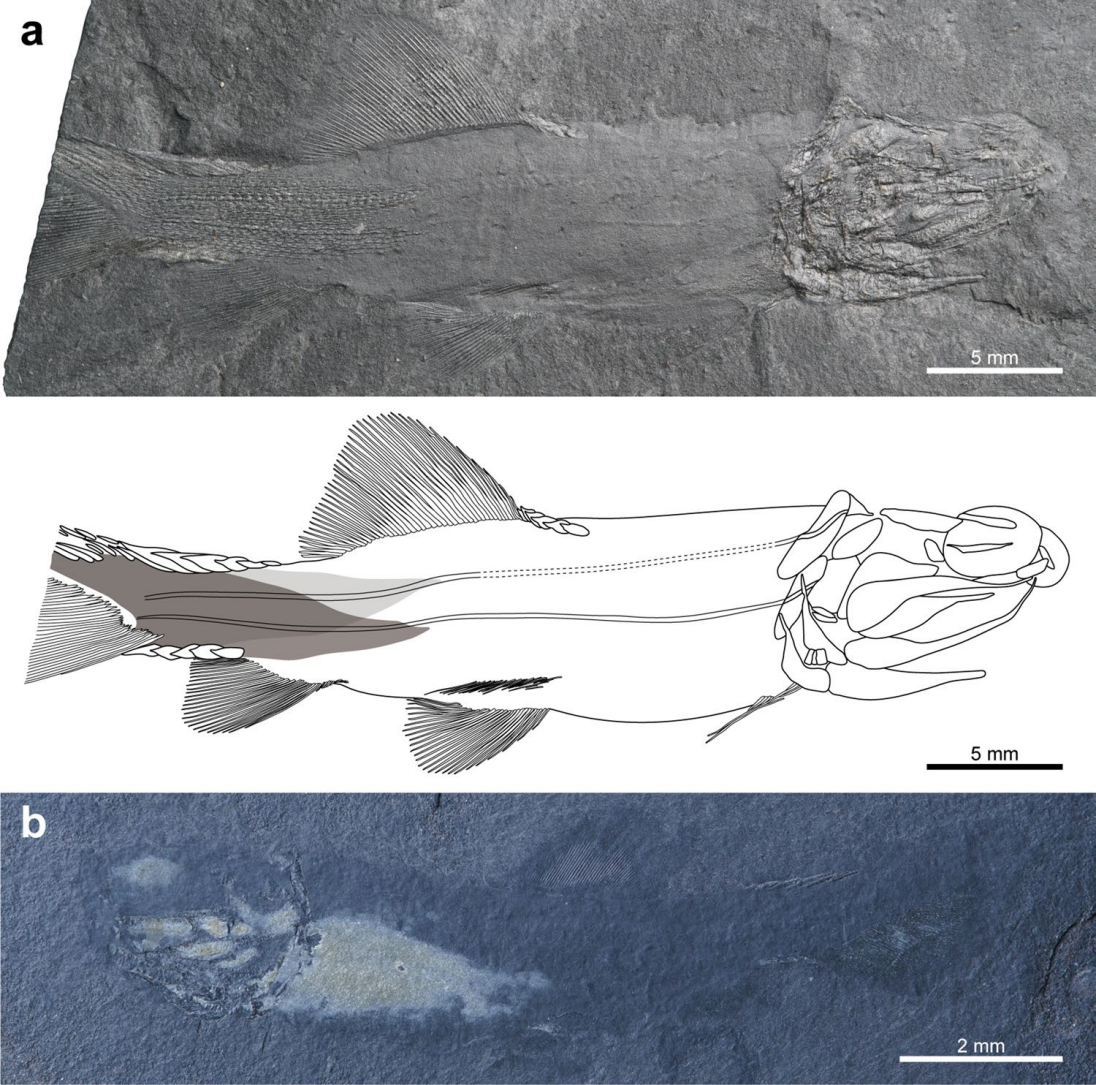
Photographs and line drawing of the holotype (GNUE11001) and paratype (BCM2016) of *Megalomatia minima* gen. et sp. nov. (**a**) Holotype, GNUE11001. (**b**) Paratype, BCM2016. Dark and light grey areas indicate right and left squamations, respectively. Dashed lines indicate reconstructed portions.

GNUE11001, a nearly complete, laterally compressed specimen, missing the distal portion of a caudal fin (Fig. 2a). The specimen was collected from Myeongcheon-dong, Boryeong.

#### Paratype

BCM2016, a nearly complete, laterally compressed specimen (Fig. 2b). The specimen is the smallest in SL and was collected from Hwasan-dong, Boryeong.

#### Referred specimens

BCM2014-1 (Supplementary Fig. S1), BCM2014-2 (Supplementary Fig. S2), BCM2017-1 (Supplementary Fig. S3), BCM2017-2 (Supplementary Fig. S4), BCM2018 (Supplementary Fig. S5), BCM2020 (Supplementary Fig. S6), BCM2021 (Supplementary Fig. S7), laterally compressed specimens; BCM2016-2 (Supplementary Fig. S8), BCM2022-1 (Supplementary Fig. S9), BCM2022-2 (Supplementary Fig. S10), BCM2023-1 (Supplementary Fig. S11), BCM2023-2 (Supplementary Fig. S12), dorsoventrally compressed specimens. The specimens were collected from Hwasan-dong, Boryeong.

#### Localities and horizon

Amisan Formation, Upper Triassic at Myeongcheon-dong and Hwasan-dong, Boryeong, South Chungcheong Province, South Korea (Fig. 1).

#### Diagnosis

*Megalomatia minima* is distinguished from other basal actinopterygians by the following combination of characters (autapomorphies with an asterisk): elongate fusiform body; large protruded eyes*; large gape with sharp teeth; oblique suspensorium; oval operculum that is much smaller than the suboperculum; trunk lateral line canals underlying the lateral line scales*; large dorsal fin opposite the pelvic fin in the middle of the body; dorsal scutes preceding the dorsal fin; a well-developed series of dorsal and ventral scutes with following basal fulcra on the caudal peduncle; strongly heterocercal caudal fin.

### Description

**Figure 3:**
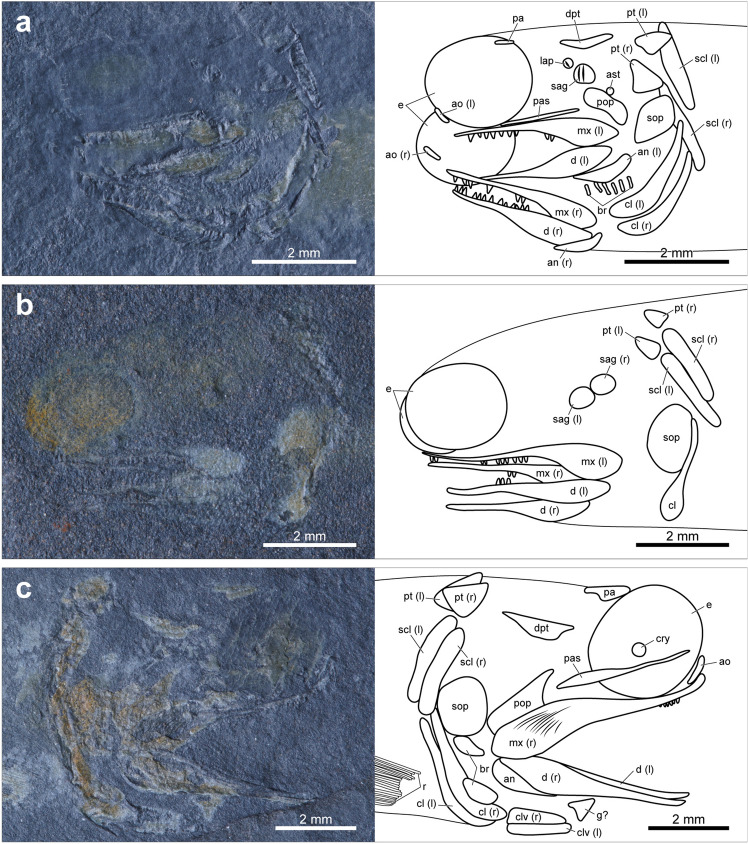

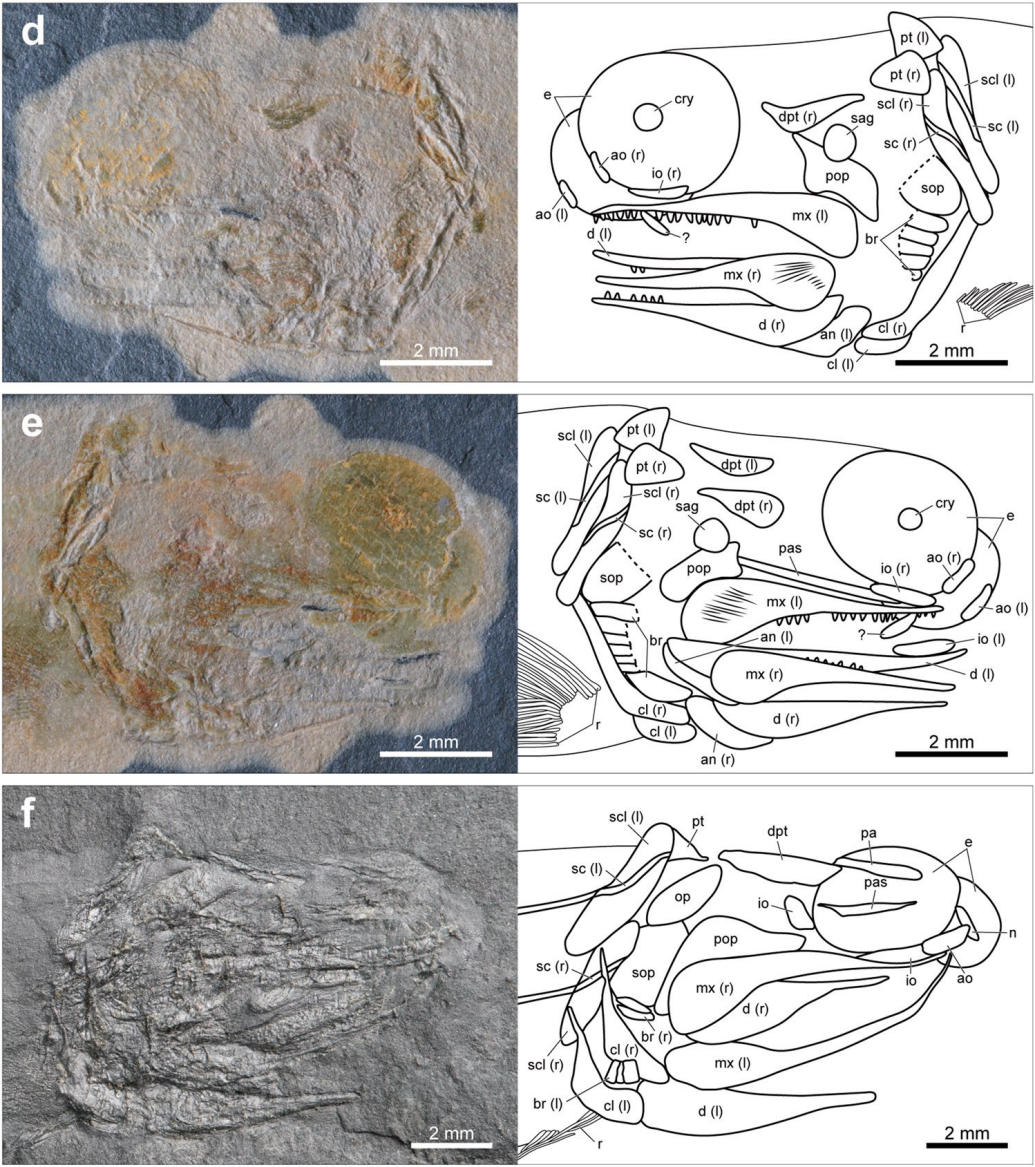
Photographs and line drawings of cranial elements of the laterally preserved specimens of *Megalomatia minima* gen. et sp. nov. The specimens are arranged in order of SL. (**a**) Paratype, BCM2016 (the smallest specimen in SL). (**b**) BCM2017-1. (**c**) BCM2021. (**d**) BCM2014-1. (**e**) BCM2014-2. (**f**) Holotype, GNUE11001 (the largest specimen in SL). *ao* antorbital, *an* angular, *ast* asteriscus, *br* branchiostegal rays, *cl* cleithrum, *clv* clavicle, *d* dentary, *dpt* dermopterotic, *e* eyes, *g?* gular?, *io* infraorbital, *(l)* left, *lap* lapillus, *mx* maxilla, *n* nasal, *op* operculum, *pa* parietal, *pas* parasphenoid, *pop* preoperculum, *pt* posttemporal, *r* pectoral fin rays, *(r)* right, *sag* sagitta, *sc* canal of supracleithrum, *scl* supracleithrum *sop* suboperculum.

Most of the specimens of *Megalomatia* are nearly complete except for one specimen (BCM2016-2, Supplementary Fig. S8), and their size series ranges from 17.8 to 33.7 mm in SL (Supplementary Table S1). Along with their small body size, the immature characters of *Megalomatia* (not fully formed cranial elements, absent or incompletely developed body squamation, and absence of yolk sac) indicate that all specimens of *Megalomatia* are in the juvenile phase. In the paratype, which is the most miniature specimen in SL (Figs. 2b, 3a), the parietal, dermopterotic, otoliths, antorbital, parasphenoid, maxilla, dentary, angular, preoperculum, soboperculum, branchiostegal rays, posttemporal, supracleithrum, cleithrum, and all fins except for the pectoral fin are present. Subsequently, as *Megalomatia* grows, the infraorbital, clavicle, nasal, posterior infraorbital, operculum, pectoral fin, and squamation appear. There is no remarkable change in body proportions as it grows.

### Cranial elements

On the skull roof of *Megalomatia*, only the parietal^20,21^ and dermopterotic are present (Fig. 3). A thin and rod-shaped parietal first appears posterior to the mid-dorsal margin of the eye and anteriorly extends as it grows (Fig. 3a,c,f).

The dermopterotic is triangular-shaped (Fig. 3a,c–f). It covers the mid-lateral portion of the skull roof, and its posterior arm extends posteriorly.

**Figure 4:**
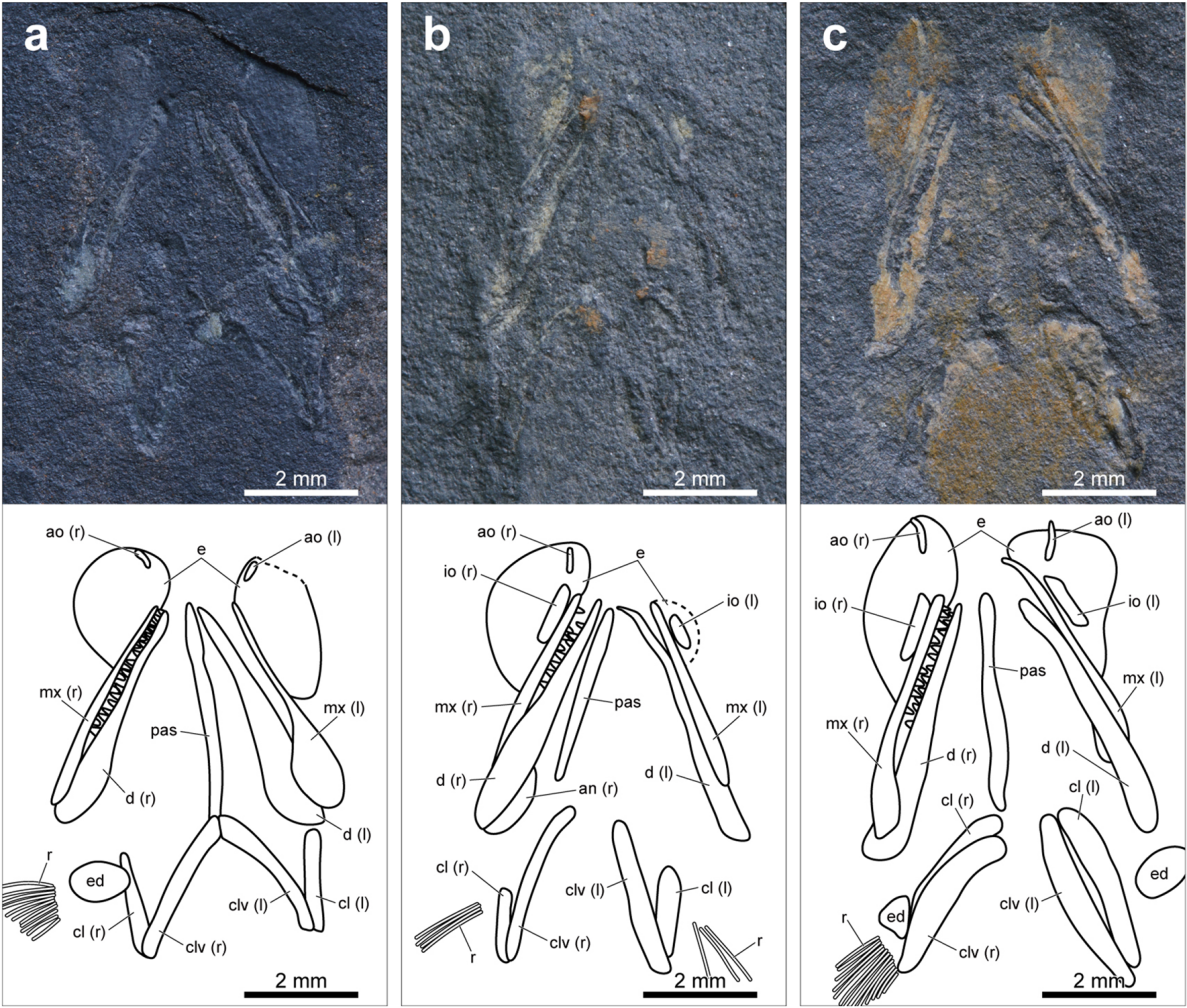
Photographs and line drawings of cranial elements of the dorsoventrally preserved specimens of *Megalomatia minima* gen. et sp. nov. (**a**) BCM2016. (**b**) BCM2022-1. (**c**) BCM2023-1. Dashed lines indicate reconstructed portions. *ao* antorbital, *an* angular, *cl* cleithrum, *clv* clavicle, *d* dentary, *e* eyes, *ed* endoskeletal disc, *io* infraorbital, *(l)* left, *mx* maxilla, *pas* parasphenoid, *r* pectoral fin rays, *(r)* right.

Based on the eye residues preserved in all specimens (Fig. 3) except for BCM2018, it is possible to identify the morphological features of the eyes of *Megalomatia*. Remarkably, the volume of the eyes can be identified due to the dorsoventrally preserved specimens (Fig. 4). The quite large eyes are oval and protrude anteriorly. The width of the eyes is more than a third of the length of the head length (from the tip of the snout to the posterior end of the suboperculum) (Fig. 3). The lateral thickness of the eyes reaches nearly half of their width (Fig. 4). Based on the position of the circumorbital series in the dorsoventrally preserved specimens (Fig. 4), approximately half of the eye would have been externally exposed. An eyelid would have covered the remaining portion.

In BCM2021, BCM2014-1, and BCM2014-2, the trace of a possible crystalline lens is preserved. This small round-shaped structure is located at the center of the eye (Fig. 3c–e).

The eyes of *Megalomatia* are surrounded by the nasal, antorbital, and infraorbitals (Fig. 3f). Because these bones are not fully formed at this developmental stage, only their approximate shape is discernable. The nasal is slightly rhombic-shaped and covers the mid-anterior margin of the eye (Fig. 3f). It contacts posteroventrally with the antorbital. The antorbital is trapezoidal and slightly deep dorsoventrally (Fig. 3f). The infraorbital is as deep as the antorbital but longer than the antorbital (Fig. 3f). The posterior portion of the antorbital and the entire infraorbital overlie the anterior arm of the maxilla. A single trapezoidal posterior infraorbital is located posterior to the eye (Fig. 3f). Although it does not entirely cover the posterior margin of the eye, it is unknown whether additional infraorbital will be formed.

The parasphenoid is discernable in the dorsoventrally preserved specimens and several laterally preserved specimens through the eyes (Figs. 3, 4). The parasphenoid is narrow and rod-shaped. It originates between the symphyses of the upper jaw and extends nearly to the level of the anterior tip of the clavicles (Figs. 3, 4). The anterior portion of the parasphenoid tapers anteriorly (Fig. 3c) and is slightly stout in dorsoventral view (Fig. 4).

The opercular series of *Megalomatia* is anteriorly inclined (Fig. 3f). The preoperculum of *Megalomatia* is hatchet-shaped (Fig. 3). The posterodorsal corner of the preoperculum is dorsally convex (Fig. 3f). The preoperculum has a slightly concave anterior margin with the anterior arm extended anterodorsally (Fig. 3c,d). In the holotype, the preoperculum covers nearly half of the dorsal margin of the maxilla (Fig. 3f). The narrow oval-shaped operculum is much smaller than the suboperculum (Fig. 3f). The suboperculum is broad and oval-shaped (Fig. 3). In the early developmental stages, the suboperculum is nearly vertically arranged and becomes anteriorly inclined as it grows.

Six branchiostegal rays are preserved below the angular in the paratype (Fig. 3a). The number of branchiostegal rays dorsally increases as it grows, but the exact number of them remains unknown because of their incomplete preservation (Fig. 3a,d–f).

The maxilla is quite long, having a large gape (Figs. 3, 4). The dorsal margin of the maxilla is nearly straight without a convex posterodorsal process and a distinct recession where the maxilla is in contact with the circumorbital bones. The maxilla has a posteroventral process, slightly covering the posterodorsal portion of the dentary (Figs. 3, 4). The anterior arm of the maxilla strongly tapers anteriorly and extends nearly to the level of the anterior margin of the eye (Fig. 3). Its anterior two-thirds of the oral margin bears evenly arranged small teeth (Figs. 3, 4). The teeth are spike-like, and their height is *c*. 0.11 mm. The surface of the maxilla is ornamented with thin rugae, which are horizontally arranged and nearly parallel to each other (Fig. 3c–e).

The dentary is straight and tapers anteriorly (Figs. 3, 4). It also bears small teeth on the anterior two-thirds of the oral margin, as in the maxilla. The angular is roughly crescent-shaped and covers the posteroventral margin of the dentary (Figs. 3a,c–e, 4b).

In the specimens of *Megalomatia*, the otoliths in situ are present between the eye and opercular region (Fig. 3a,b,d,e). Remarkably, the paratype exhibits exceptional preservation of the otoliths, including a lapillus, sagitta, and asteriscus (Fig. 3a). The lapillus, which is located most anterior position, is rounded and has an anteriorly inclined ridge. The sagitta, commonly observed in the specimens of *Megalomatia*, is relatively large. Two ridges with a sulcus between them are located on the anterior portion of the sagitta and are nearly perpendicular to the long axis of the body (Fig. 3a). The asteriscus is slightly distant from the other otoliths. It is tiny and lacks a distinct structure.

### Postcranial elements

The pectoral girdle of *Megalomatia* is among the first parts of the development process (Figs. 3, 4). The posttemporal is triangular-shaped with a concave anterior margin, and its posterior edge contacts the supracleithrum (Fig. 3a). The supracleithrum is relatively narrow, and its dorsal and ventral margin is rounded (Figs. 3, 4). A prominent sensory canal is preserved on each supracleithrum in the holotype (Fig. 3f). The canals are inclined anteriorly with a slight curve and in contact posteriorly with each side of the trunk lateral line canal. The cleithrum is longer than the supracleithrum and curved gently (Figs. 3, 4). The dorsal arm of the cleithrum extremely tapers, having a pointed tip, while the ventral arm is short and stout (Fig. 3f). There is no notch on the posteroventral margin of the cleithrum for the pectoral fin insertion (Figs. 3, 4). The clavicles are elongated and triangular-shaped in lateral view and are posteriorly in contact with the cleithrum (Figs. 3c, 4). In ventral view, a pair of clavicles exhibits a rod-like shape and meets each other in the middle anteriorly (Fig. 4).

**Figure 5:**
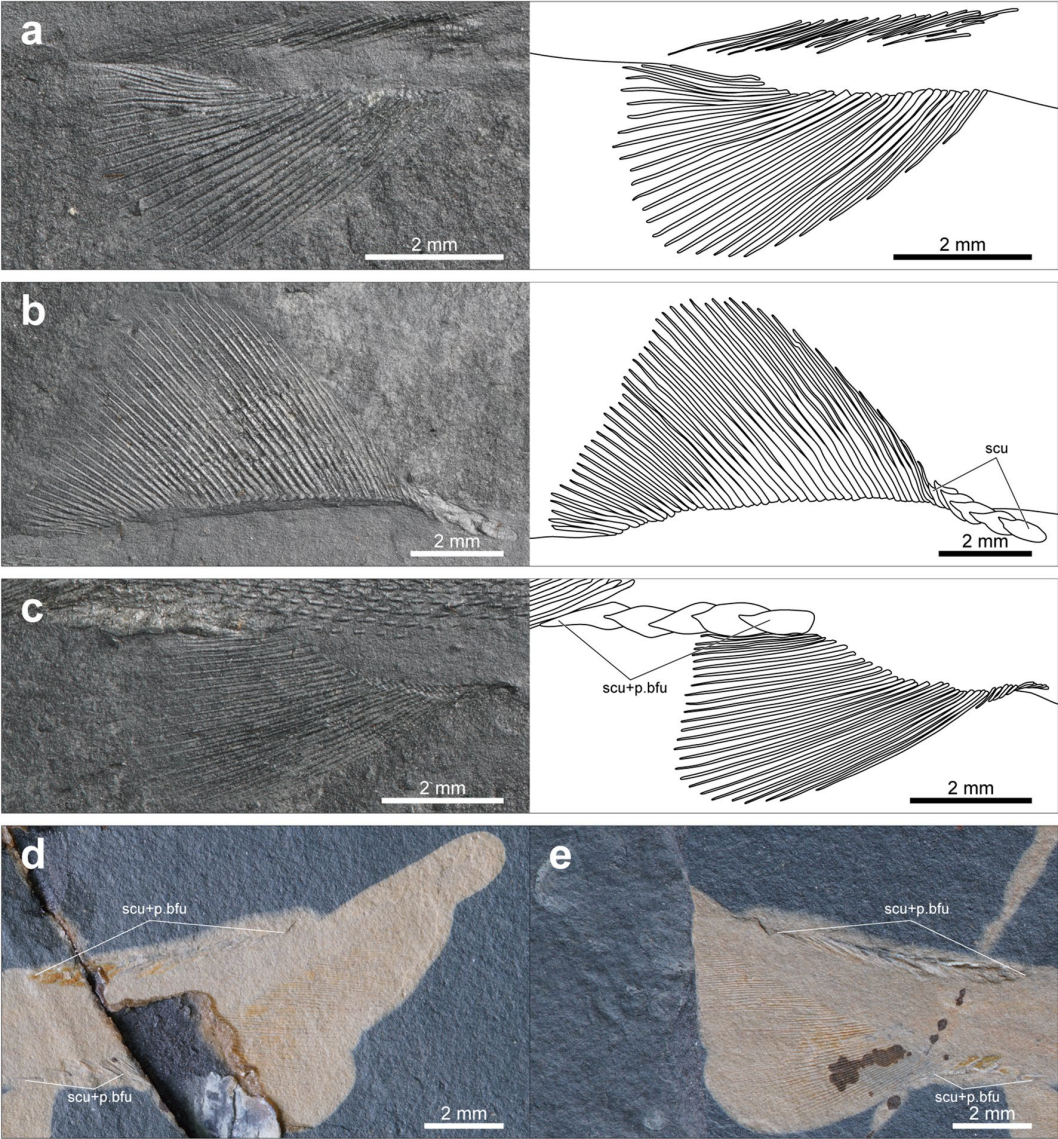
Photographs and line drawings of pelvic, dorsal, anal, and caudal fins of *Megalomatia minima* gen. et sp. nov. (**a**) Pelvic fin of the holotype (GNUE11001). (**b**) Dorsal fin of the holotype (GNUE11001). (**c**) Anal fin of the holotype (GNUE11001). (**d**) Caudal fin of BCM2014-1. (**e**) Caudal fin of BCM2014-2. *p.bfu* paired basal fulcra, *scu* scutes.

The fin rays of both paired and median fins are neither segmented nor branched, and fringing fulcra, which are paired lanceolate structures associated with the leading rays of fins^22^, are absent (Figs. 2, 5). Due to the small size of the specimens and the incomplete development of their fins, counting the exact number of fin rays is difficult. However, the number of fin rays tends to increase throughout the fin development, although there is some individual variation, and it is not the highest in the holotype, which is in the latest developmental stage (Supplementary Table S1). The pterygiophores of the median fins are absent even in the holotype, while the endoskeletal disc of the pectoral fin, which has not yet undergone differentiation into radials, can be discernable between the cleithrum and pectoral fin rays (Fig. 4a,c).

The pectoral fin is located close to the ventral margin of the body (Figs. 2, 3c–f). It is the last fin to appear among all fins and is discernable starting with the specimen measuring 24.3 mm in SL (BCM2021, Fig. 3c). Approximately 23 fin rays are present in BCM2014-1, which exhibits the best-preserved pectoral fin among the specimens of *Megalomatia* (Fig. 3e).

The pelvic fin is located at the mid-length of the ventral margin of the body and is closer to the anal fin origin than to the pectoral fin (Fig. 2). The pelvic fin consists of 28 fin rays in the holotype (Fig. 5a). The pelvic girdle is not observed in all specimens of *Megalomatia*.

The dorsal fin is located at the mid-length of the dorsal margin of the body and in near opposition to the pelvic fin (Fig. 2). However, the pelvic fin originates slightly anterior to the dorsal fin origin. The dorsal fin is prominently large and consists of 45 fin rays in the holotype (Fig. 5b). The dorsal fin base is almost twice that of the pelvic fin (Figs. 2, 5b,c). The dorsal margin of the dorsal fin is rounded. Six scutes precede the dorsal fin only in the holotype (Fig. 5b).

The anal fin originates ventrally directly posterior to the end of the dorsal fin. The exact number of anal fin rays of the holotype remains unknown due to incomplete preservation of its anterior portion (Figs. 2, 5c). However, the anal fin of *Megalomatia* tends to have approximately ten fewer rays than the dorsal fin (Supplementary Table S1).

The caudal fin is strongly heterocercal, and the dorsal lobe extends beyond the end of the caudal fin (Figs. 2, 5d,e). The caudal fin consists of 81 fin rays in BCM2014-1 and its counterpart specimen, BCM2014-2, which exhibit the best-preserved caudal fin among the specimens of *Megalomatia* (Fig. 5d,e). The caudal fin forms a slightly S-shaped border with the margin of the caudal peduncle (Fig. 2a). In the relatively early developmental stages, the posterior margin of the caudal fin is rounded without any curves (Fig. 2b). Throughout the caudal fin development, its posterior margin gradually becomes S-shaped by having a concave portion along the longitudinal axis of the body (Fig. 5d).

A series of the scutes with the following paired basal fulcra covers the dorsal and ventral margin of the caudal peduncle (Figs. 2, 5d,e). A series of the dorsal scutes and basal fulcra is located at the midpoint between the end of the dorsal fin and the posterior tip of the caudal fin when they first appeared (Fig. 2b). The number of them increases both forward and backward direction throughout growth (Supplementary Table S1, Figs. 2a, 5d,e). The five ventral scutes and basal fulcra cover entirely the whole ventral margin of the caudal peduncle in the holotype (Figs. 2a, 5c). Their number also increases throughout growth (Supplementary Table S1).

**Figure 6:**
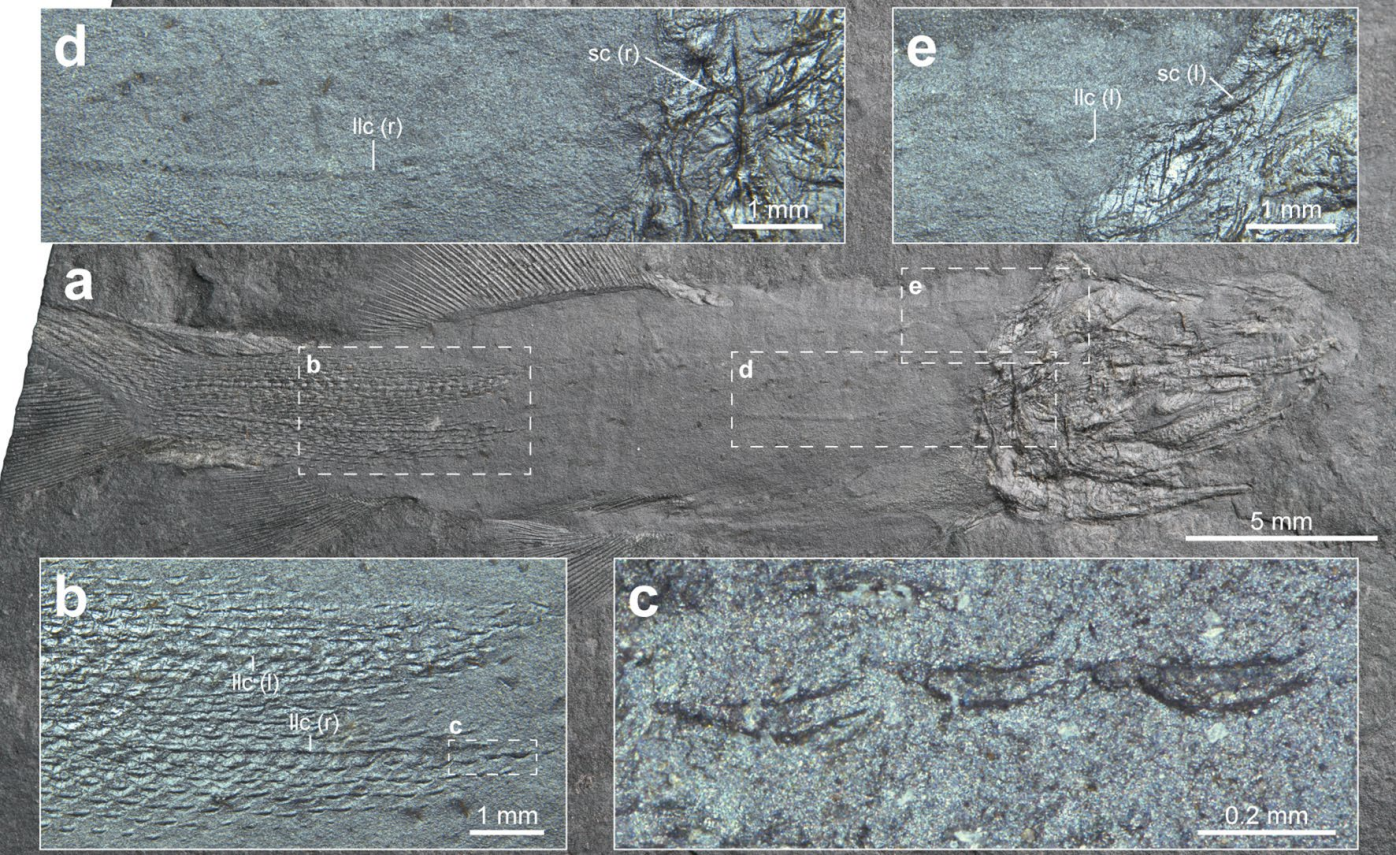
Photographs of lateral line canals and squamations of the holotype (GNUE11001) of *Megalomatia minima* gen. et sp. nov. (**a**) Holotype, GNUE11001. (**b**) Right and left squamations. (**c**) Magnified scales from (**b**). (**d**) Right lateral line canal with a canal of right supracleithrum. (**e**) Left lateral line canal with a canal of left supracleithrum. *(l)* left, *llc* lateral line canal, *(r)* right, *sc* a canal of supracleithrum.

The incomplete squamation is only present in the holotype and BCM2018, exhibiting the early development of the squamation (Fig. 6, Supplementary Fig. S6). In the holotype, both the right and left sides of the squamation are preserved (Fig. 6b). The squamation pattern originates on the posterior portion of the caudal fin and extends anteriorly to the level of the middle of the dorsal fin. In particular, the scale rows along each trunk lateral line and the adjacent rows develop earlier than the other rows. The scale rows that are closer to the dorsal and ventral margin of the body develop relatively late so that the squamation has a wedge-shaped anterior margin (Fig. 6b). The tiny scales are needle-shaped, and their length is *c*.0.3 mm (Fig. 6c). The scales are regularly distributed along the longitudinal rows and do not entirely fill the gaps between each scale due to their small size.

Remarkably, both the right and left sides of the trunk lateral line canal are preserved in the holotype (Fig. 6d,e). Although it looks as if two canals are present on the right side of the trunk, they are continuously connected to the sensory canal on each side of the supracleithrum. On the caudal peduncle, the trunk lateral line canal runs under the scales instead of passing through them (Fig. 6b). Thus, the scale row overlying the canal is slightly raised and more conspicuous than the other rows.

## Discussion

Several preserved features of the specimens of *Megalomatia* indicate that they are in the juvenile phase, including not fully formed cranial elements, absent or incompletely developed body squamation, and absence of yolk sac (Figs. 2, 3, 4, 6). The ontogenetic information of *Megalomatia* provides valuable insight into the developmental patterns of basal actinopterygians and the adaptations of young individuals to enhance their viability under primitive body plans.

**Figure 7:**
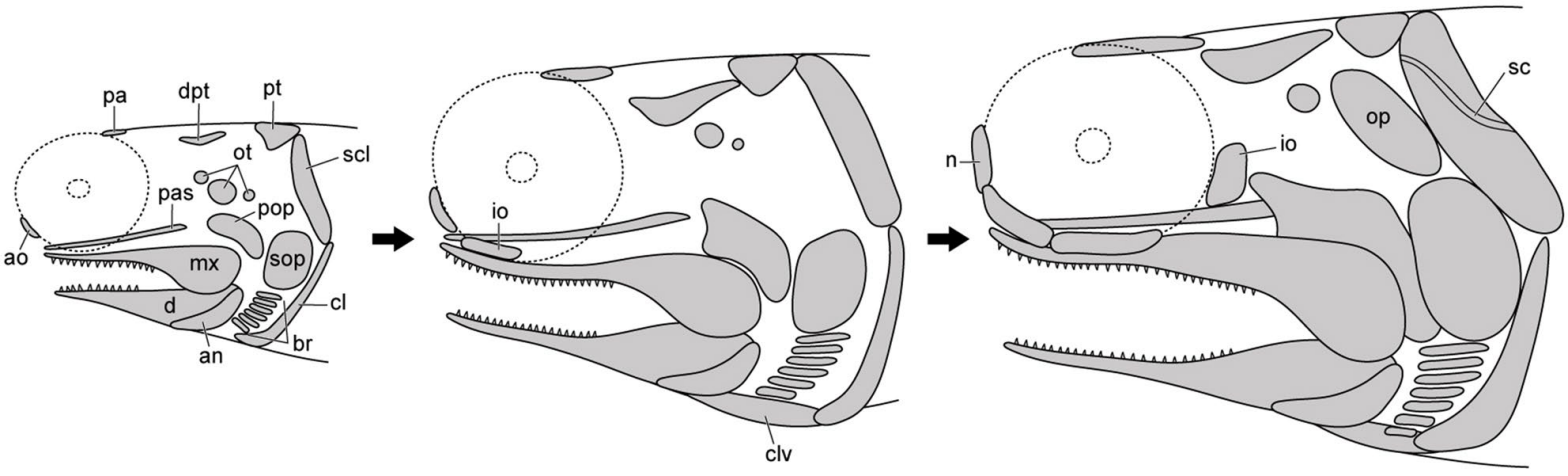
Development of cranial elements of *Megalomatia minima* gen. et sp. nov. The arrows indicate the direction of ontogenetic change. *ao *antorbital, *an* angular, *br* branchiostegal rays, *cl* cleithrum, *clv* clavicle, *d* dentary, *dpt* dermopterotic, *io* infraorbital, *mx* maxilla, *n* nasal, *op* operculum, *ot* otoliths, *pa* parietal, *pas* parasphenoid, *pop* preoperculum, *pt* posttemporal, *sc* a canal of supracleithrum, *scl* supracleithrum, *sop* suboperculum.

Based on the specimens of *Megalomatia*, we can observe the gradual development of the cranial and postcranial elements within the growth series of *Megalomatia* (Figs. 3, 7) with a focus on specific elements, which develop first to appear. The parietal and dermopterotic of *Megalomatia* appear first in the development of the skull roof, preceding the other bones. In general, the parietal is among the first bones to form during the skull roof development, and the dermopterotic follows it^23–25^. The development of the orbit of *Megalomatia* begins with the antorbital covering the anteroventral portion (Figs. 3, 7), followed by the appearance of the infraorbital—subsequently, the nasal and posterior infraorbital cover the anterior and posterior margins of the eye, respectively. In the opercular series of *Megalomatia*, the operculum is the last bone to appear, following the preoperculum and suboperculum (Figs. 3, 7). This developmental pattern differs from that of modern fishes, where the appearance of the operculum precedes the other bones of the opercular series^25^. The preoperculum initially exhibits an elongated oval shape (Fig. 3a). During development, it extends anteriorly and posteriorly, covering the dorsal margin of the maxilla. Its anterior margin gradually becomes concave, and its posterodorsal corner becomes dorsally convex (Figs. 3c–f, 7). In the holotype (Fig. 3f), the concave anterior margin of the preoperculum is not clearly discernable. It appears that the anteroventral portion of the preoperculum is covered by the maxilla, revealing only the extended anterior arm. Unlike most of the basal actinopterygians, which generally possess the segmented and branched fin rays with the fringing fulcra, these characteristics are not observed in all specimens of *Megalomatia* (Fig. 5). In the early development of the fin rays, the degree of segmentation of fin rays is low, and the borders of each segment are not distinct compared to that of mature individuals^24^. Throughout the growth of fins, characteristics such as distally added segments, bifurcated fin rays, and fringing fulcra developing along the anterior margin of the fins are observed^24^. Therefore, considering the developmental process of the fins, the absence of segmentation, bifurcation, and fringing fulcra in the fin of *Megalomatia* seems to be a temporary feature observed only during early developmental stages.

Although all specimens of *Megalomatia* represent its juvenile phase, they exhibit some characteristics that can be used to assign *Megalomatia* as ‘palaeoniscoids’, such as the anteriorly inclined opercular series and a hatchet-shaped preoperculum. The anteriorly inclined opercular series and suspensorium are commonly observed in most of the ‘palaeoniscoids’ although there is some variation of inclination angle^26^. A hatchet-shaped preoperculum is also commonly used to describe ‘palaeoniscoids’^26^. Meanwhile, *Megalomatia* has an operculum much smaller than the suboperculum. Such a small operculum is observed in a few ‘palaeoniscoid’ taxa, including *Canobius* and *Cheirodopsis*^27^.

Due to the exceptional preservation of the specimens of *Megalomatia*, we were able to confirm the presence of the possible crystalline lens, the otoliths, and the trunk lateral line canals (Figs. 3, 6), which are generally difficult to observe on the fish fossils. The crystalline lenses of aquatic animals tend to be much rounder and harder than those of terrestrial animals to provide focusing ability by having enough refractive power^28^. In particular, fish lenses are very hard due to the high protein concentration in the lens’s center^29^. Therefore, considering the hardness of fish lenses and the size and shape of the preserved structure at the center of the eye of *Megalomatia* (Fig. 3c–e), it is reasonable to consider this structure as the crystalline lens. The possible crystalline lens is also recognizable in a specimen of *Hiascoactinus*^16^.

In the paratype (BCM2016, Fig. 3a), three types of otoliths (lapillus, sagitta, and asteriscus) are preserved. The slight spacing and difference in morphology and size between them indicate that each represents a different type of otolith rather than a pair of the same type. Furthermore, their relative position corresponds to the actual arrangement of the otoliths within the inner ear (lapillus, sagitta, and asteriscus from the front)^30,31^. The largest and smallest otolith of *Megalomatia* are the sagitta and asteriscus, respectively, as in most extant and extinct fishes^31,32^. The formation of otoliths occurs in very early developmental stages^23,24^, so it is possible to estimate fish age. However, the fossil record of basal actinopterygians with all three types of otoliths in situ is extremely scarce. Gottfried^33^ described two large otoliths, which were identified as the lapillus and sagitta, and a tiny third one, identified as an asteriscus, in a ‘palaeoniscoid’ specimen. However, Coates^34^ reinterpreted two large ones as a pair of asterisci and the third one as sagitta and suggested that a small sagitta and large asteriscus, contained within a single sacculo-lagenar chamber, are primitive characteristics for basal actinopterygians. In *Megalomatia*, however, the sagitta is the largest among the preserved otoliths with a straight sulcus, which is a typical feature of sagitta^32^. Therefore, discovering three types of otoliths in *Megalomatia* supports Gottfried’s interpretation, and Coates’s hypothesis should be reconsidered.

**Figure 8:**
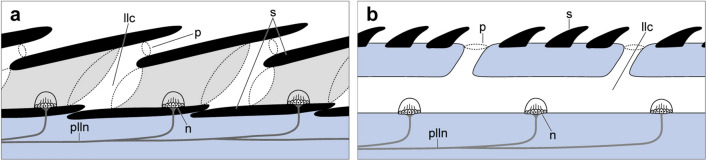
Lateral line canal of modern actinopterygians and chondrichthyans. (**a**) Lateral line canal of modern actinopterygians (modified from Webb and Ramsay^59^). Note that the lateral line canal runs through the scales. Light grey areas indicate canal segments. (**b**) Lateral line canal of chondrichthyans (modified from Hickman et al.^71^). Note that the lateral line canal runs under the skin. *llc* lateral line canal, *n* canal neuromast, *p* pore, *plln* posterior lateral line nerve, *s* scale.

In the teleosts, the formation of the trunk lateral line canal generally occurs in concert with the development of the scales^35,36^. During the development of lateral line scales, the lateral walls, arising from the scale plate, form an enclosed tube. The lateral line canal passes through the tubes on the overlapping lateral line scales^37^ (Fig. 8a). In contrast, in the holotype of *Megalomatia*, the prominent trunk lateral line canals are observed in the anterior trunk region, where the squamation has not yet developed, and continue posteriorly under the lateral line scales on the caudal peduncle (Fig. 6). It suggests that the development of the trunk lateral line canal of *Megalomatia* precedes that of the squamation rather than simultaneously. Furthermore, assuming the presence of ossified tubes enclosing the canals seems reasonable. These ossified tubes probably help the canals retain their relief during fossilization. The trunk lateral line canal with the ossified tubes is also recorded in living basal actinopterygians (*Polyodon spathula*) and extinct basal chondrichthyans (*Falcatus falcatus*)^38,39^. Particularly, paddlefish possess rhomboid scales exclusively on the dorsal lobe of the tail, and their lateral line canals run under the scales as in *Megalomatia*. The trunk lateral line canal, which does not pass through scales and appears independently without association with scale plate, is commonly observed in acanthodians and elasmobranchs (Fig. 8b)^3,36,40–43^. Therefore, such a relationship between trunk lateral line canal and squamation development, observed in the major groups within gnathostomes, seems to represent a plesiomorphic trait of gnathostomes. The osteichthyan lineage has independently lost this trait by acquiring the canal that passes through the tubes on the overlapping lateral line scales.

The evolution of the feeding mechanism in actinopterygians begins with gradual changes in their jaw structure from a simple hinge-like jaw joint to a mouth capable of tube-like extension with mobile jaw elements^44^. In the advanced teleosts, several anatomical features, such as an elongated ascending process on the premaxilla that makes the premaxilla slide anteriorly, the maxilla that can swing anteriorly, and the laterally expandable suspensorium are major adaptations for the suction feeding mechanism^44–46^. Using these structures, fishes rapidly expand their mouth cavity, creating a drop in pressure and drawing the prey and water encompassing it into the mouth^44–46^. In basal actinopterygians, however, the firmly attached maxilla and premaxilla to the other dermal skull bones, the anteriorly inclined suspensorium that limits the lateral expansion of the mouth cavity, and a large gape that disturbs the maintenance of low pressure in the mouth cavity are inappropriate for suction feeding. Instead, as a bite feeder, they would have used ram feeding to approach the prey and seize it. Additionally, a large gape and small pointed teeth probably were well-suited to seize the large prey. As in other basal actinopterygians, *Megalomatia* shows these primitive conditions in the skull elements (Figs. 3, 4), and it supports that *Megalomatia* would also have used this basal feeding mode.

Due to the small suction power, bite feeders draw less prey toward their mouth and require more movement toward the prey during a feeding event^47^. For this reason, bite feeding would have required a more precise-targeting strategy to seize the prey than suction feeding, and the large protruded eyes of *Megalomatia* are considered a possible adaptation for feeding success under the condition of the primitive jaw structure. Increased eye size is correlated with enhanced visual acuity, and it is reported across many species, including fishes, birds, water fleas, and marine reptiles^48–53^. The larger the eye is, the more retinal photoreceptor cells it accommodates. Thus, the retinal photoreceptor cells increase in number and receive more light per solid angle of image space, and a better resolution of an image is obtained^48,49,54,55^. Furthermore, the smaller inter-receptor angle corresponding to the enlarged eye leads to higher acuity^52,54,56^.

As the feeding strategy has evolved to suction feeding, the snout region tends to be anteriorly extended to accommodate the specialized jaw elements, and the eyes are located posteriorly relative to the mouth. Consequently, the advanced teleosts have a narrow binocular visual field, which rarely exceeds 40°^55,57^, and a blind area is formed directly anterior to the snout. In contrast, the protruded and more frontally placed eyes of *Megalomatia* suggest that there is no tradeoff between the advanced feeding strategy and the advantage in the visual field. These distinctive eyes of *Megalomatia* would have provided a wide binocular field without the blind area. It probably results in the enhanced perception of depth and the range of the prey and the ability to visually track the elusive prey, even when it is located directly anterior to the snout until the feeding process is completed.

The large eyes of *Megalomatia* can be considered a result of allometric growth in early developmental stages. However, the body proportions in the juvenile phase are generally similar to those of adult individuals^5,58^. Thus, the large protruded eyes of *Megalomatia* are considered a diagnostic morphological feature of *Megalomatia* rather than a temporary feature that only appears in early developmental stages. Meanwhile, an enormous and anteriorly placed orbit is commonly observed in basal actinopterygians, including *Belichthys*, *Lineagruan*, and *Pteronisculus*^11,59,60^. It possibly suggests that the condition of the eyes with wide binocular vision, similar to *Megalomatia*, may not be restricted to *Megalomatia* and would have been a vital adaptation to successfully catch the prey in basal actinopterygians before acquiring the ability of suction feeding. However, further discoveries of specimens with clear eye residues are required to reveal the morphological features and functions of the eyes of basal actinopterygians.

Although there is some variation in the degree of formation and preservation of skull bones, the upper and lower jaws, pectoral girdle, and opercular series consistently appear first through all specimens (Fig. 3). These elements are essential for feeding and respiration. The jaws with sharp teeth and pectoral girdle, where the sternohyoideus muscle that opens the mouth is attached, play an essential role in capturing their prey. The opercular series protects the gill complex, and water movement through the buccal and opercular cavities, necessary for respiration, is facilitated by the opening and closing of the opercular series^61^. As the yolk sac is resorbed in modern fishes, young individuals switch from relying on endogenous nutrient sources to eating exogenous food. Additionally, they decrease their reliance on cutaneous respiration and instead increase their use of branchial respiration^62^. Likewise, the preferential formation of the jaws, pectoral girdle, and opercular series in the specimens of *Megalomatia* is likely an adaptation resulting from the same survival requirements after the resorption of the yolk sac.

Generally, the squamation development in basal actinopterygians initiates anteriorly and progresses posteriorly^5,7,8,59^. A few scale rows quickly cover more than half of the lateral line, and the following additional scale rows develop both above and below the lateral line, exhibiting a narrow wedge-shaped squamation margin. Subsequently, the squamation also occurs on the dorsal lobe of the tail and combines with the squamation from the anterior part of the caudal peduncle as it grows. Whereas, *Megalomatia* exhibits some difference in the squamation development. The squamation of the *Megalomatia* appears first on the caudal region and extends anteriorly along the lateral line (Fig. 6b). The development of the lateral line scale row slightly precedes the other scale rows, exhibiting relatively thick wedge-shaped squamation margin. Such a posteroanterior squamation pattern has been reported in *Parhaplolepis* (‘palaeoniscoids’), acanthodians, and living chondrichthyans and osteichthyans^63–66^. Remarkably, the dorsal and ventral scutes with the following paired basal fulcra on the caudal peduncle appear to occur before the squamation development in *Megalomatia* (Figs. 2b, 5d, e) as well as in basal actinopterygians, where the squamation development has been reported such as *Elonichthys* and *Brookvalia*^7,8^. The early appearance of the squamation on the caudal region would have increased the stiffness of the tail, and the stiffer tail improves the swimming capability of fish by increasing thrust force and swimming speed^6,67–69^. Furthermore, before the appearance of the squamation, the presence of the scutes and basal fulcra, arranged along the dorsal and ventral margin of the caudal peduncle, probably increased the stiffness of the tail with a lower cost in a much shorter time. It suggests that the acquisition of swimming capability during early developmental stages is an adaptation for chasing prey and evading predators, which are essential for the survival of young individuals of basal actinopterygians, including *Megalomatia*.

Meanwhile, the wide binocular visual field of *Megalomatia* decreases the monocular visual field, leading to an increase in the posterior blind area behind the head. Consequently, it suggests that the tail of *Megalomatia* falls within the posterior blind area, making it vulnerable to possible damage. The fish tail is a vital appendage where the propulsive thrust reaches its maximum^70^. The primary function of the scales is to protect the vital and vulnerable parts. Thus, it is reasonable to consider that the scales, which initially appear on the caudal region of *Megalomatia*, also play a significant role in protecting its tail.

## Conclusions

Fourteen specimens from the Upper Triassic Amisan Formation of South Korea represent a new taxon, *Megalomatia minima* gen. et sp. nov. Their exceptional preservation provides ontogenetic information and insight into possible adaptations in response to functional demands to increase their viability. The primitive arrangement of actinopterygian otoliths has been the subject of debate. However, the discovery of the otoliths in situ of *Megalomatia* does not support the hypothesis that the presence of the asteriscus as the largest one among the three otoliths represents the primitive condition for basal actinopterygians. The trunk lateral line canal of *Megalomatia* appears independently without association with the scale plate and runs under the scales. This characteristic is observed in the major groups within gnathostomes and seems to represent a plesiomorphic gnathostome trait. As a bite feeder with a primitive jaw structure, *Megalomatia* would have required a more precise targeting strategy to seize the prey. The large protruded eyes of *Megalomatia* probably provided a wide binocular field without the anterior blind area and played a significant role in targeting and catching prey. In all specimens of *Megalomatia*, the upper and lower jaws, pectoral girdle, and opercular series consistently appear first. The preferential formation of these skeletal elements would have allowed juvenile *Megalomatia* to obtain nutrients from exogenous food and increase their branchial respiration after the yolk sac resorption. Furthermore, the squamation of *Megalomatia* firstly covering the caudal region would have improved the swimming capability by increasing the tail stiffness and played a role in protecting its tail from possible damage. All these characteristics of *Megalomatia*, appearing in the juvenile phase, are likely linked to the adaptation of young individuals to increase their viability.

### Supplementary Information

Supplementary Information.

## Data Availability

All data generated or analyzed during this study are included in this published article and its Supplementary Information files.
