# Supplementary material for: A new exceptionally well-preserved basal actinopterygian fish in the juvenile stage from the Upper Triassic Amisan Formation of South Korea
Source: Sci Rep. 2024 Jan 3;14:317. doi: 10.1038/s41598-023-50803-z (PMC10764774; doi:10.1038/s41598-023-50803-z)
Supplement: Supplementary file 1 — Supplementary Information. [file 41598_2023_50803_MOESM1_ESM.docx]

**Supplementary Information**

**A new exceptionally well-preserved basal actinopterygian fish in the juvenile stage from the Upper Triassic Amisan Formation of South Korea**

Su-Hwan Kim^1^, Yuong-Nam Lee^1^*, Gi-Soo Nam^2^, Jin-Young Park^3^, Sungjin Lee^1^, Minyoung Son^4^

^1^ School of Earth and Environmental Sciences, Seoul National University, Seoul, 08826, South Korea

^2^ Gongju National University of Education, Gongju, South Chungcheong Province, 32553, South Korea

^3^ Gwacheon National Science Museum, Gwacheon-si, Gyeonggi-do, 13817, South Korea

^4^ Department of Earth and Environmental Sciences, University of Minnesota Twin Cities, Minneapolis, Minnesota, United States

**Contents**

1. Supplementary Figures

2. Supplementary Table

**1. Supplementary Figures**


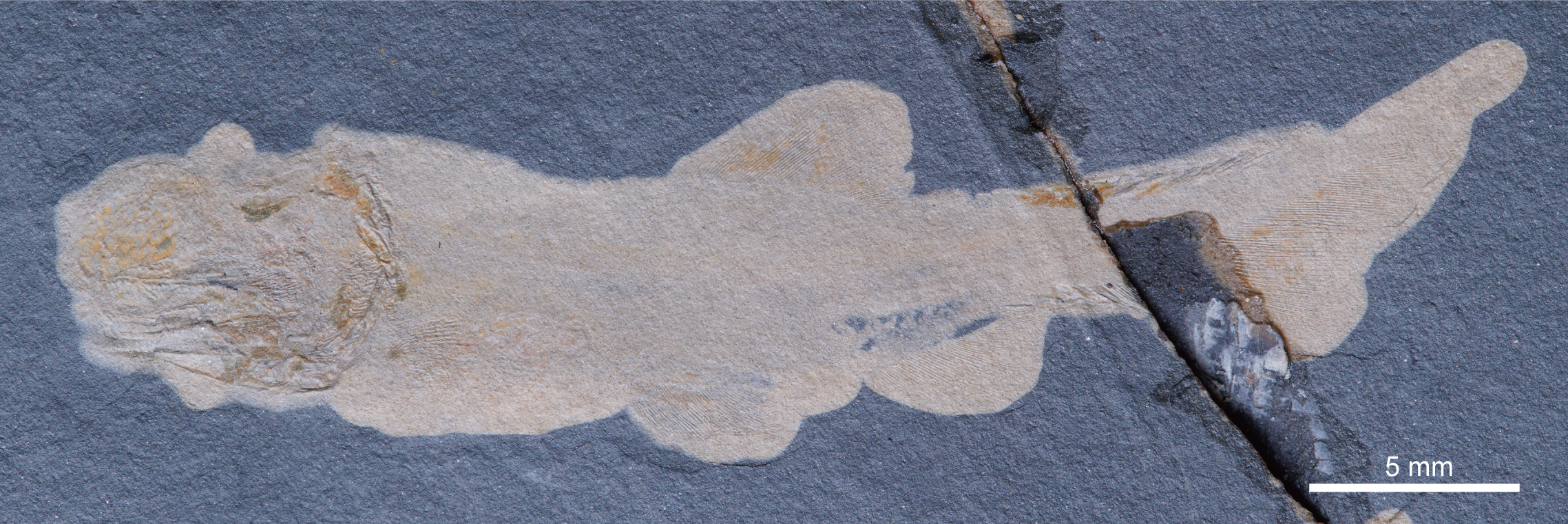


**Supplementary Figure S1.** BCM2014-1, *Megalomatia minima* gen. et sp. nov.


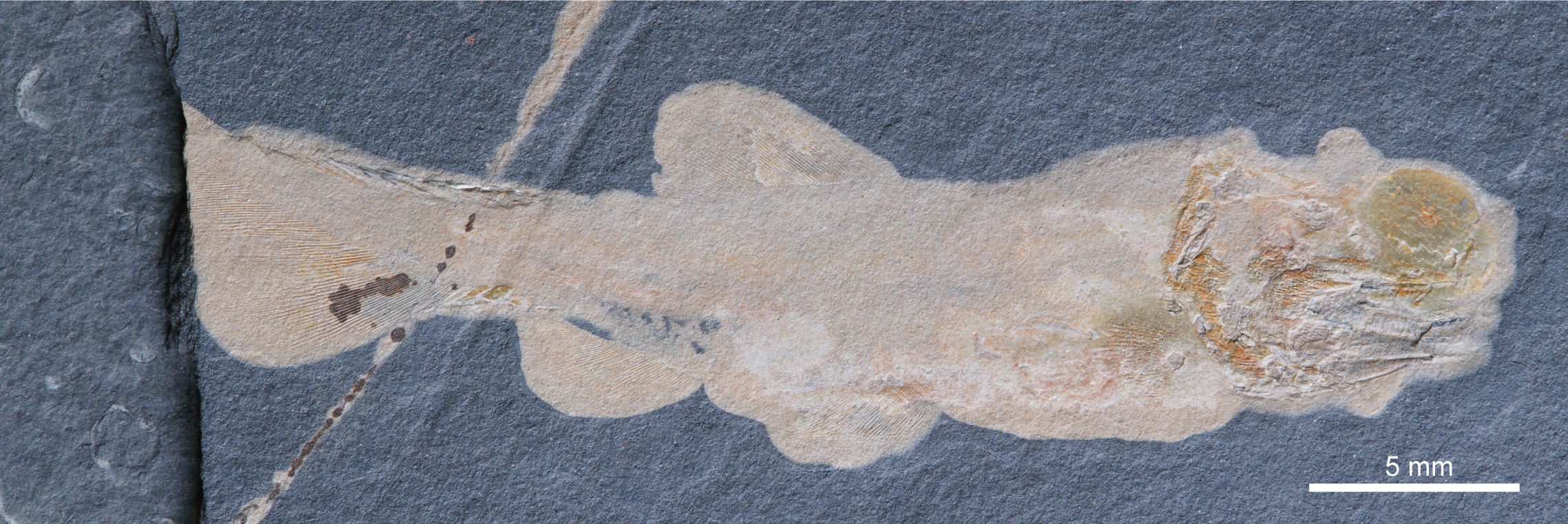


**Supplementary Figure S2.** BCM2014-2, *Megalomatia minima* gen. et sp. nov.


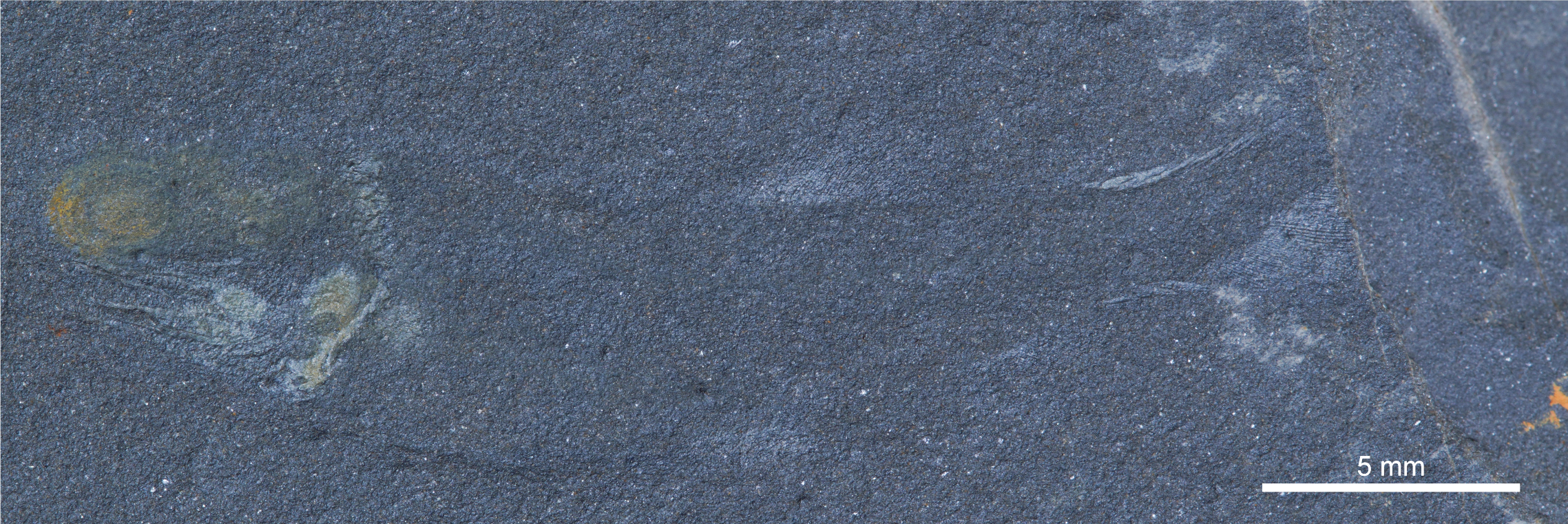


**Supplementary Figure S3.** BCM2017-1, *Megalomatia minima* gen. et sp. nov.


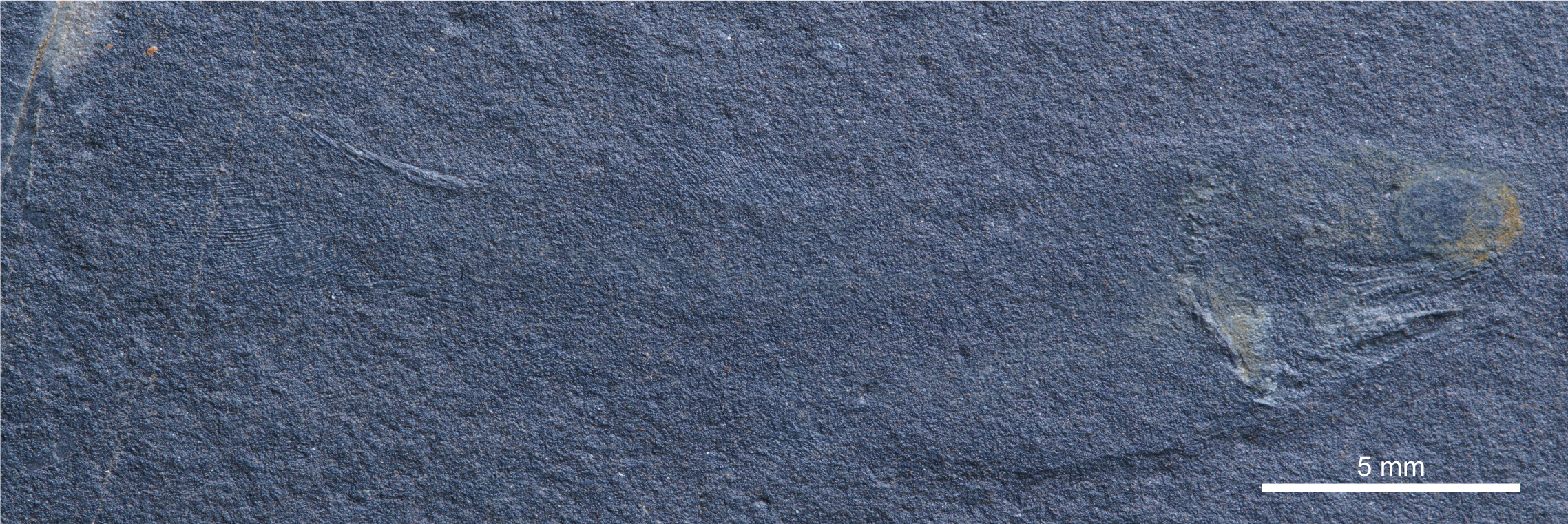


**Supplementary Figure S4** BCM2017-2, *Megalomatia minima* gen. et sp. nov.


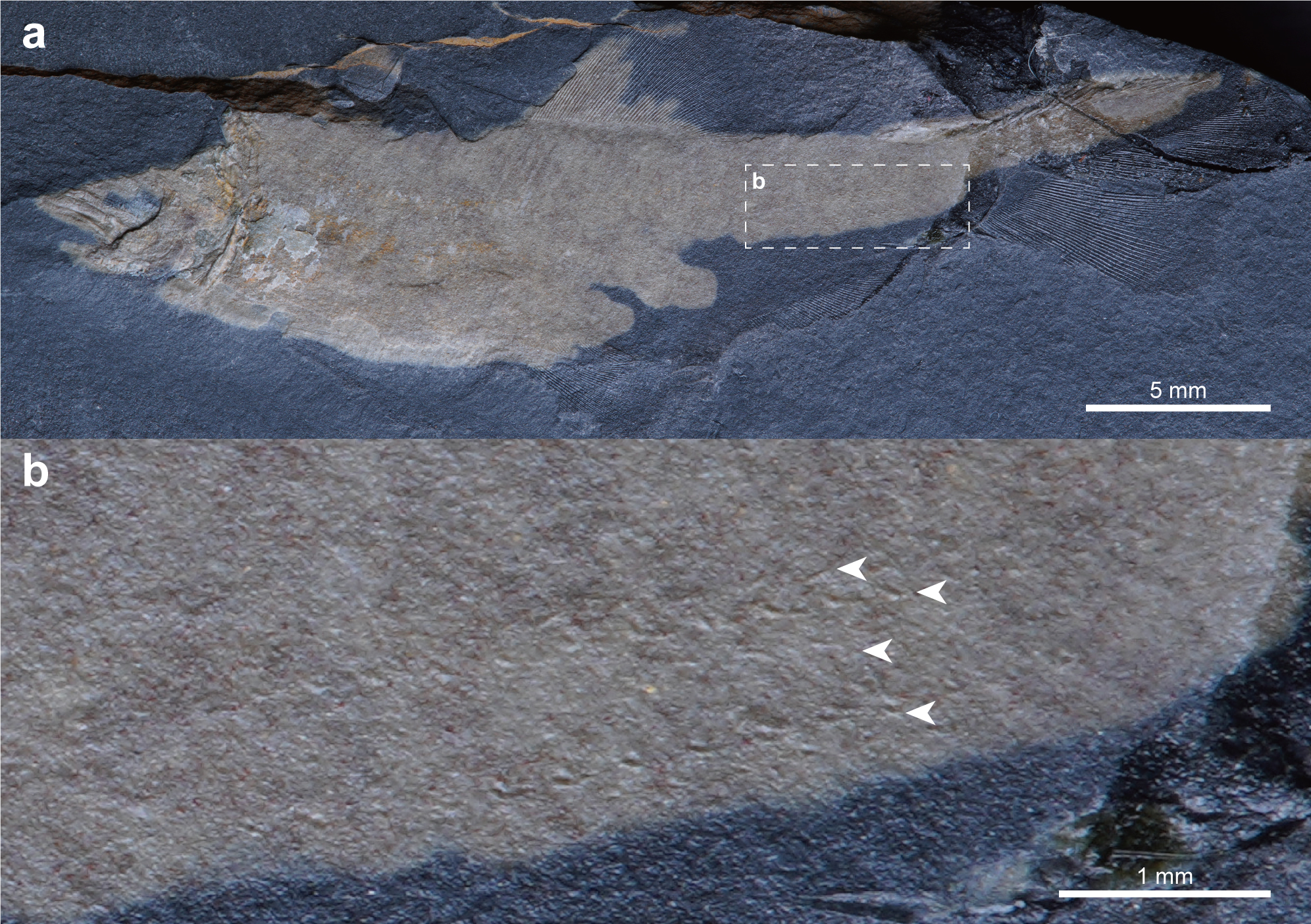


**Supplementary Figure S5.** BCM2018, *Megalomatia minima* gen. et sp. nov. (a) Photograph of BCM2018, *Megalomatia minima* gen. et sp. nov. (b) Magnified scales from (a). White arrows indicate scale rows.


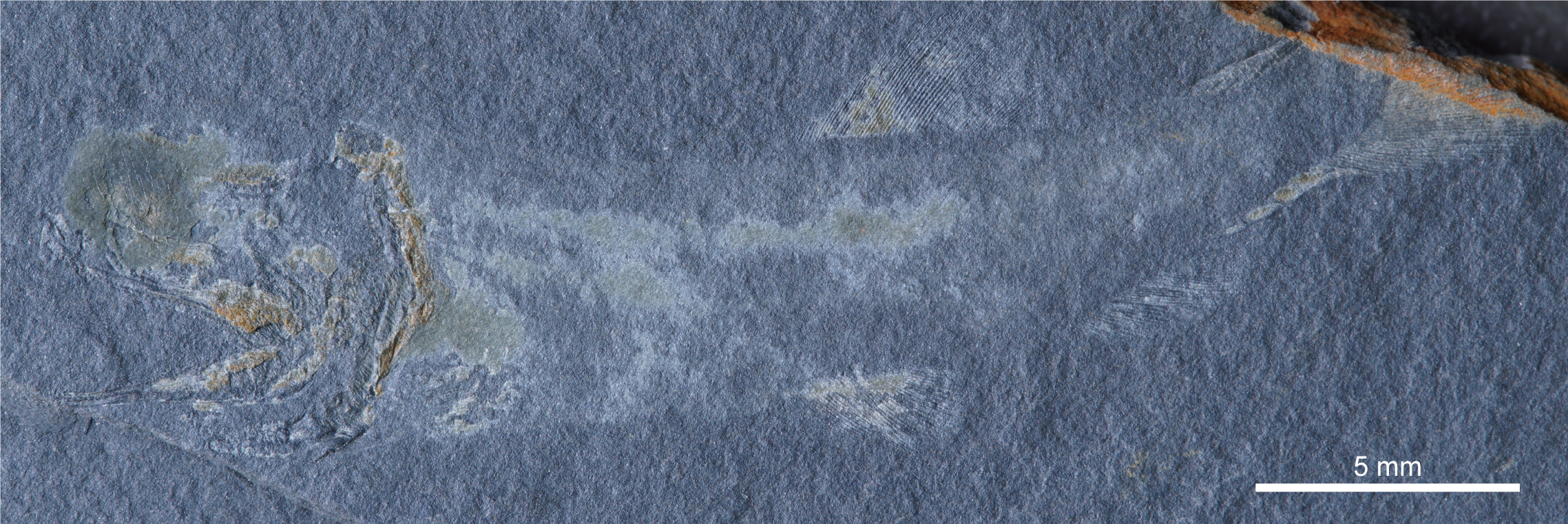


**Supplementary Figure S6.** BCM2020, *Megalomatia minima* gen. et sp. nov.


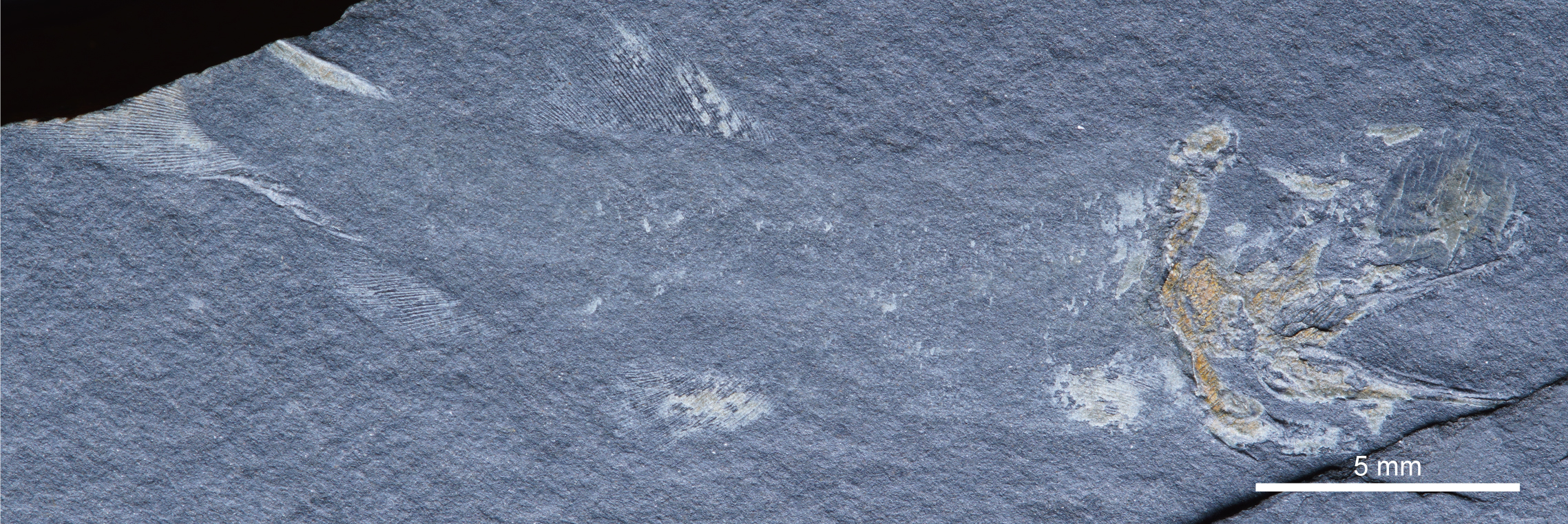


**Supplementary Figure S7.** BCM2021, *Megalomatia minima* gen. et sp. nov.


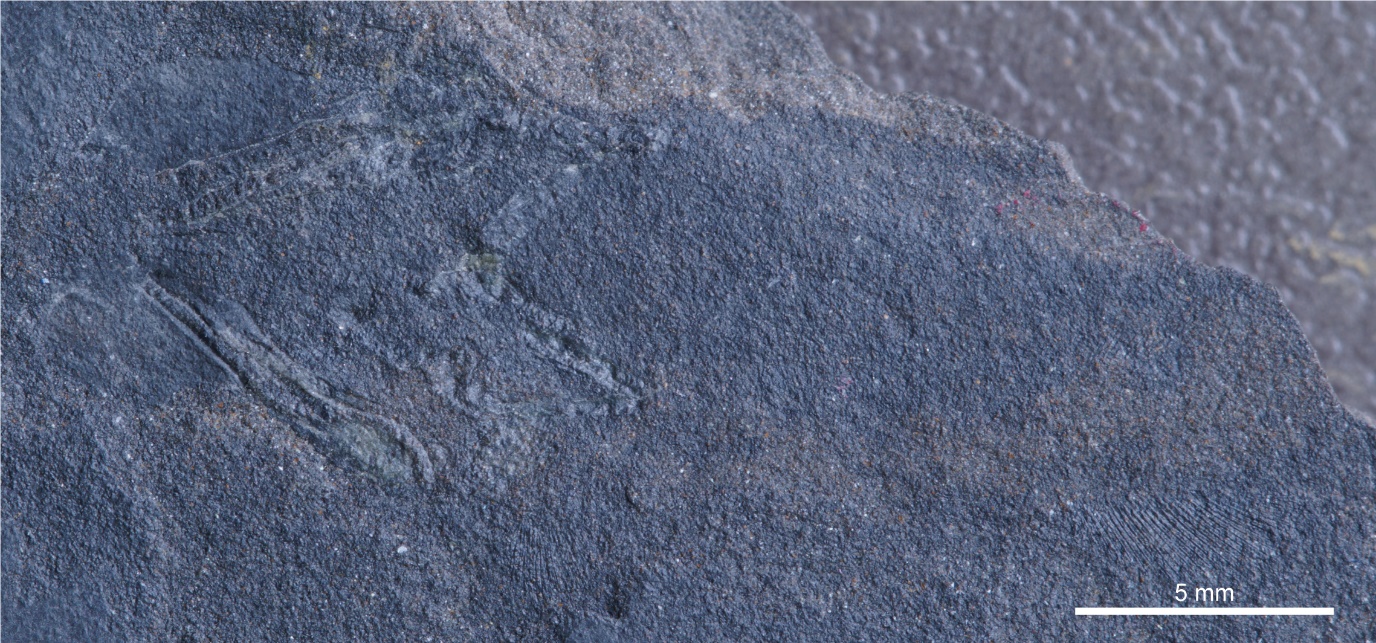


**Supplementary Figure S8.** BCM2016-2, *Megalomatia minima* gen. et sp. nov.


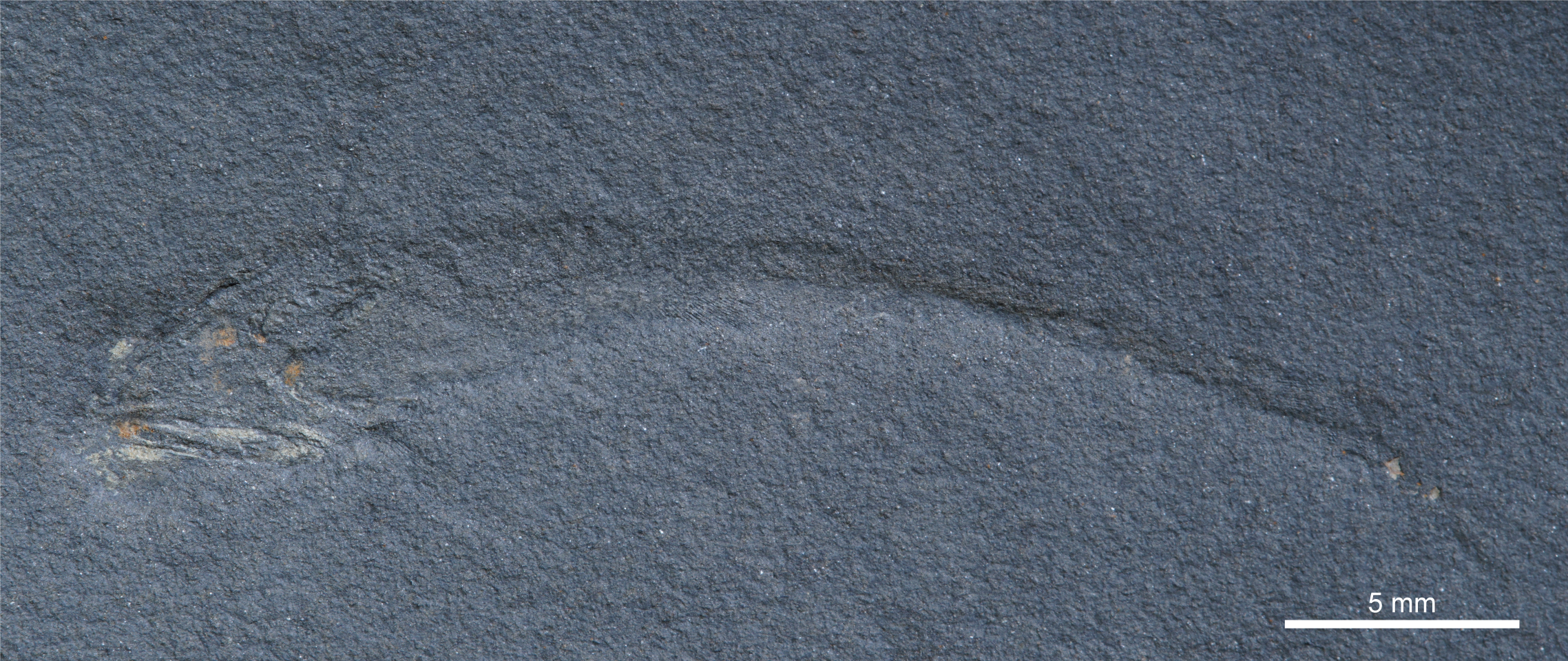


**Supplementary Figure S9.** BCM2022-1, *Megalomatia minima* gen. et sp. nov.


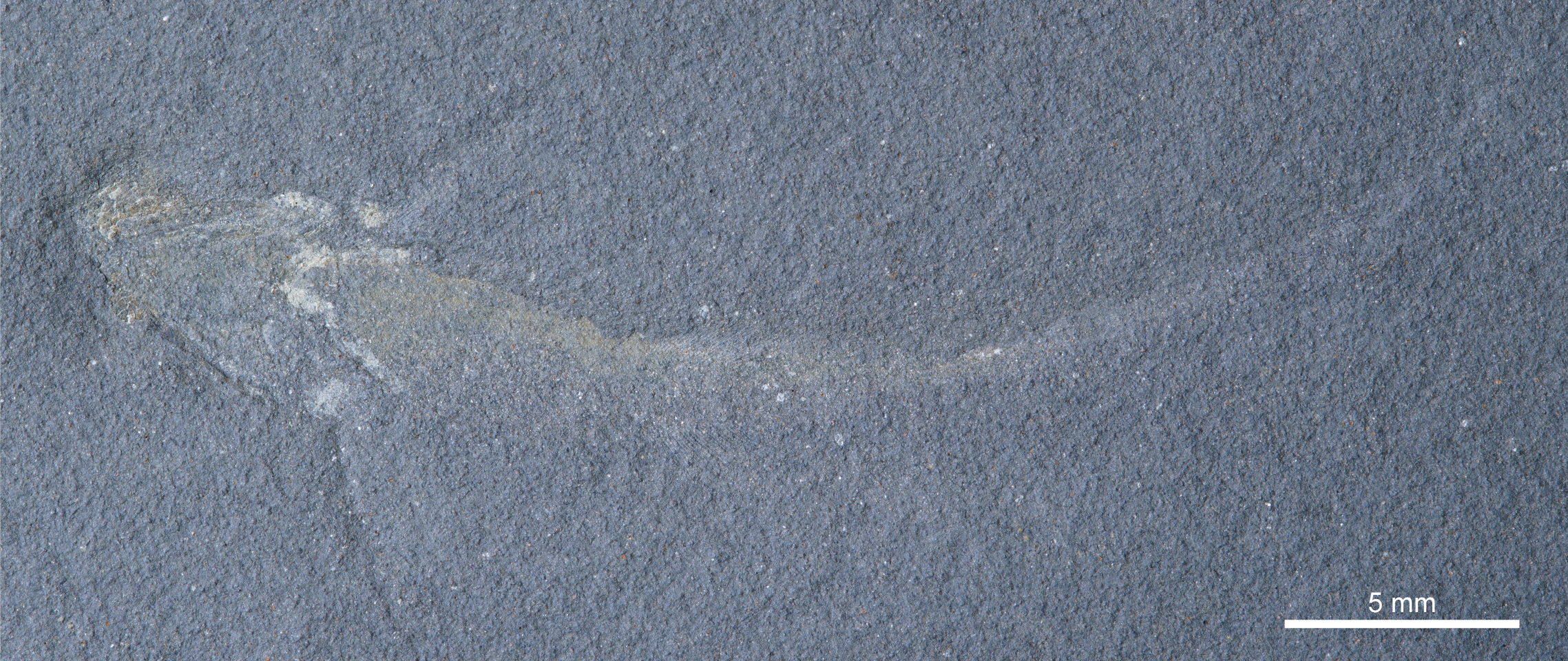


**Supplementary Figure S10.** BCM2022-2, *Megalomatia minima* gen. et sp. nov.


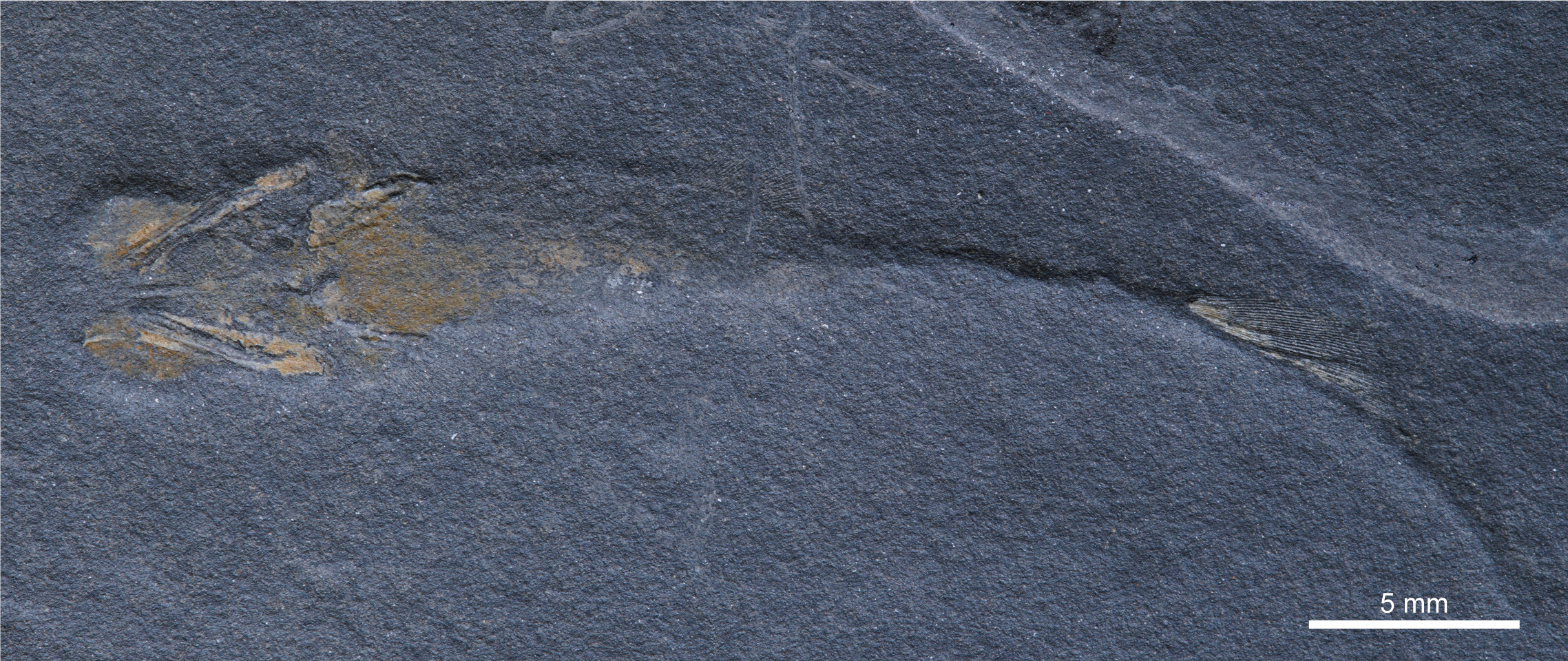


**Supplementary Figure S11.** BCM2023-1, *Megalomatia minima* gen. et sp. nov.


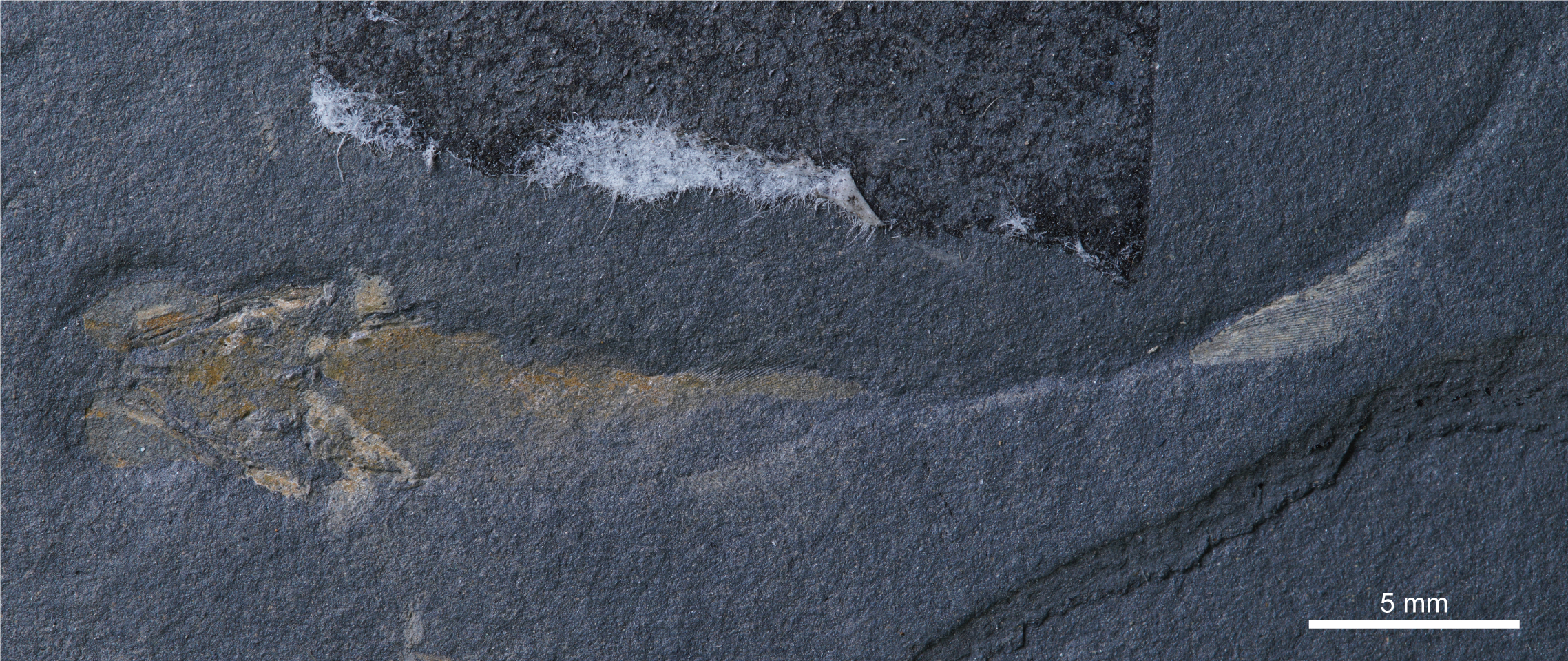


**Supplementary Figure S12.** BCM2023-2, *Megalomatia minima* gen. et sp. nov.

**2. Supplementary Table**

**Supplementary Table S1.** Meristic measurements of the specimens of *Megalomatia* *minima* gen. et sp. nov.

| Measurements | BCM2016  (paratype) | BCM2017-1  BCM2017-2 | BCM2020  BCM2021 | BCM2014-1  BCM2014-2 | BCM2018 | BCM2023-1  BCM2023-2 | BCM2022-1  BCM2022-2 | GNUE11001  (holotype) | BCM2016-2 |
| --- | --- | --- | --- | --- | --- | --- | --- | --- | --- |
| Total length  (mm) | 21.9 | 28.8 | ? | 34.9 | ? | 36.2 | ? | ? | ? |
| Standard length  (mm) | 17.8 | 22.2 | 24.3 | 25.1 | 25.5 | 26.0 | 26.6 | 33.7 | ? |
| Eye length (mm) | 2.1 | 2.2 | 2.8 | 2.9 | ? | 3.3 | 2.8 | 3.6 | 2.6 |
| Branchiostegal rays | 6 | ? | ? | 7 | ? | ? | ? | ? | ? |
| Pectoral fin rays | ? | ? | ? | *c*. 23 | ? | ? | ? | ? | ? |
| Pelvic fin rays | 14 | ? | ? | *c*. 32 | *c*. 28 | *c*. 28 | ? | 28 | ? |
| Dorsal fin rays | 41 | *c*. 40 | *c*. 46 | *c*. 56 | *c*. 54 | ? | ? | 45 | ? |
| Anal fin rays | 27 | ? | *c*. 30 | *c*. 44 | *c*. 44 | ? | ? | ? | ? |
| Caudal fin rays | 58 | *c*. 60 | ? | *c*. 81 | *c*. 80 | ? | ? | ? | ? |
| Dorsal scutes + paired basal fulcra | 3 + 3 | 6 | ? | 12 | *c*. 17 | ? | ? | 7 + ? | ? |
| Ventral scutes + paired basal fulcra | 2 + 1 | 3 | 5 | 3 + 2 | 5 | ? | ? | 5 | ? |

The specimens with their counterpart specimens (when present) are arranged in order of standard length. Standard length refers to the horizontal distance from the tip of the snout to the level of ventral origin of the caudal fin. ? indicates uncertainty due to incomplete development or preservation. In cases where the distinction between the scutes and paired basal fulcra is unclear, the sum of their number is recorded.
